# Effects of Carbon Fiber Content on the Crystallization and Rheological Properties of Carbon Fiber-Reinforced Polyamide 6

**DOI:** 10.3390/polym16172395

**Published:** 2024-08-23

**Authors:** Jianglin Liu, Lang He, Dongdong Yang, Jianguo Liang, Runtian Zhao, Zhihui Wang, Xiaodong Li, Zhanchun Chen

**Affiliations:** 1 College of Mechanical and Vehicle Engineering, Taiyuan University of Technology, Taiyuan 030024, China; 15035422561@163.com (L.H.); 19935440611@163.com (D.Y.); liangjianguo20@tyut.edu.cn (J.L.); tyutzrt@163.com (R.Z.); 17703408774@163.com (Z.W.); lxd15713535671@163.com (X.L.); chenzhanchun2003@163.com (Z.C.); 2 Advanced Metal Composite Forming Technology and Equipment Ministry of Education Engineering Research Center, Taiyuan University of Technology, Taiyuan 030024, China

**Keywords:** carbon fiber, polyamide 6, crystallization, rheological properties

## Abstract

Carbon fiber (CF)-reinforced polyamide 6 (PA6) composites have an excellent performance, attributed to properties such as light quality, high strength, and vibration reduction, and they are widely used in fields such as aerospace and transportation. Four kinds of carbon fiber-reinforced polyamide 6 (CF/PA6) composite pellets with carbon fiber contents of 20, 30, 40, and 50 wt.% were prepared using twin screw extrusion. The results were characterized using a simultaneous thermal analyzer, capillary rheometer, electronic universal material testing machine, and scanning electron microscope (SEM); their crystallization, rheological behavior, mechanical properties, surface structure, etc., were studied. DSC results indicate that an increase in carbon fiber content enhances the thermal stability of CF/PA6 and narrows the crystallization window but has a minor effect on the molecular chain diffusion time. The crystallinity reaches its maximum at a carbon fiber content of 40 wt.%, reaching 55.16%. The steady-state rheological behavior reveals that CF/PA6 behaves as a pseudoplastic fluid, exhibiting shear-thinning behavior. When the carbon fiber content is 40 wt.%, the power law exponent (*n*) reaches its maximum, and the consistency coefficient (*K*) decreases by 300 $\mathrm{Pa}\cdot s^{n}$ compared to the 30 wt.% content. With increasing temperature, *n* increases while *K* decreases. SEM observations reveal that samples with carbon fiber contents of 20 wt.% and 40 wt.% exhibit better fiber dispersion and orientation. However, the interfacial bonding strength is superior in the 40 wt.% sample. When the carbon fiber content reaches 50 wt.%, significant injection molding defects occur at the clamping end, leading to extensive matrix tearing during tension testing.

## 1. Introduction

The application of polymer composites in both civil and military applications has become increasingly important. In addition to their excellent mechanical properties, the main reason for their widespread use is their high strength-to-weight ratio [1,2,3]. The matrix material of the traditional continuous fiber-reinforced composites is a thermoset resin, but it has the disadvantage of being difficult to recycle. However, in the context of sustainable development and the impact of waste on the environment, there is now a growing demand for more easily recyclable thermoplastic matrix composites [4,5,6] Short-fiber-reinforced thermoplastics (SFRTP), composites reinforced by short fibers that can be injection-molded, are expected to exhibit high productivity and recyclability [7,8,9]. An important representative of thermoplastic composites is polyamide 6 (PA6). Carbon fiber (CF), due to its high specific modulus, high specific strength, good electrical conductivity, corrosion resistance, and excellent thermal properties, is extensively utilized as a reinforcement phase in polymer composites. Carbon fiber (CF)-reinforced polyamide 6 (PA6) composites have an excellent performance, attributed to properties such as their light quality, high strength, and vibration reduction, and they are widely used in fields such as aerospace and transportation.

Crystallization refers to the ordered arrangement of molecular chains. Higher crystallinity corresponds to a greater proportion of crystalline regions within the polymer matrix, leading to an increased tensile strength and modulus. However, the toughness and impact strength tend to decrease accordingly [10]. Um et al. investigated the influence of crystallinity on the mechanical behavior of carbon fiber-reinforced polyethylene–terephthalate (CF/PET) composites at room temperature and high temperatures. As a result, the crystallized CF/PET composites were improved by 11.6 times in the in-plane shear strength and by 3.78 times in the shear modulus at high temperature [10]. Wang et al. studied the crystallization and melting process of PA4, a special self-nucleation phenomenon which slowed the rate of recrystallization. Their study confirmed the significant differences in the morphology of crystals generated at different temperatures [11]. Xu et al. successfully prepared a series of long-chain branched polyamide 6 samples (LCB PA6s) with well-defined branch lengths and investigated their rheological properties. The results show that the increase in branch length leads to an increase in zero-shear viscosity and storage modulus, a decrease in the loss coefficient, and a high strain-hardening coefficient, i.e., the elastic response and melt strength of PA6 are enhanced [12]. Perez-Martin et al. evaluated the effect of thermal history on crystallinity development in unreinforced PEKK and CF/PEKK. As a result, the inclusion of carbon fibers increased the proportion of secondary crystallization in the matrix and slowed down the crystallization kinetics [13]. 

The rheological properties of carbon fiber-reinforced plastics are determined by the distribution of the molecular chain segment structure, the structure of the aggregation state, and the molding process, which determines the formability and usability of the products in production. Kim et al. investigated the effects of initial fiber length and screw extrusion speed on the viscosity of a carbon fiber-reinforced polycarbonate composite system, and found that when the lower the shear rate, the longer the initial fiber length, and the greater the viscosity of the material; as the extrusion speed increased, the diameter of the fibers in the material became shorter, and the viscosity of the melt decreased [14]. Das et al. utilized a melt spinning method to extrude carbon fibers (CF) and polyamide 6 (PA6) through a heated nozzle to deposit long fibers and form parts for composites, investigating changes in rheological behavior, crystallinity, and mechanical properties. The results indicated that the addition of CF enhanced the viscosity and shear-thinning behavior of the composites. Compared to PA6 fibers alone, CF/PA6 fibers exhibited a broader crystallization window, facilitating interchain diffusion and residual stress relief, thereby enhancing the tensile strength of the composites [15]. Li et al. employed a twin screw extrusion method to manufacture CF/PA6 composites with varying carbon fiber contents and conducted dynamic rheological analysis. The findings revealed that the storage modulus and loss modulus of the materials increased with CF content. Strong bonding existed at the fiber–matrix interface, enhancing the matrix’s stress-bearing capacity. However, CF also entangled molecular chains and disrupted polymer flow, significantly reducing melt flowability [16]. Atabek Savas et al. also found that an increase in carbon fiber content increased the tensile strength and Young’s modulus of the material [17].

However, too high a content of carbon fibers can cause orientation difficulties and fiber buildup, resulting in the matrix not being able to fully infiltrate the carbon fibers, greatly reducing the overall interfacial strength and leading to molding defects, so that the composite material cannot give full play to its mechanical properties, which to a certain extent restricts its application. Therefore, it is relatively important and urgent to study the optimal carbon fiber content of composites. In this study, we prepared pelletized CF/PA6 composites with carbon fiber contents of 20, 30, 40, and 50 wt.% using twin screw extrusion and investigated the crystallization, melting behavior, rheological properties, and mechanical performance of these composites, and their fracture morphology was analyzed using SEM.

## 2. Experiment

### 2.1. Materials

Polyamide 6 (PA6), B3S, was supplied by BASF SE, Germany. PA6 has a melting temperature of about 220 °C, a tensile strength of 90 MPa, and a density of 1.13 g/cm^3^. Carbon fiber (CF), SYT49S-12K, supplied by Zhongfu Shenying Carbon Fiber Co., Ltd. (Jiangsu, China) has a tensile strength of over 4900 MPa and a density of 1.80 g/cm^3^.

### 2.2. Sample Preparation

For the tests of crystallinity and steady-state rheological properties, CF/PA6 pellets were fabricated following the steps below. First, the PA6 resin was dried in a vacuum drying oven (DZF-6020B, Shanghai Kuntian Laboratory Instrument Co., Ltd., Shanghai, China) at 110 °C for 6 h for dehydration, and after drying, it was added with carbon fibers of different strands into a twin-screw extruder (Nanjing Kolker Extrusion Equipment Co., Ltd., Nanjing, China) to mix and extrude to obtain the pellets with four different fiber contents, namely 20, 30, 40, and 50 wt.%. The screw speed was 160 r/min, the feed rate of PA6 was 2.5 Hz, and the extrusion temperature was set to five temperatures according to the roles of different zones of the screw, which were 240 °C, 245 °C, 250 °C, 245 °C, and 240 °C, respectively. After that, the resulting pellets were again put into a vacuum drying oven and dried at 110 °C for 6 h to obtain CF/PA6 pellets.

For the tensile strength test, the required tensile samples will follow the steps below. The prepared pellets were put into a vertical injection molding machine (HYD-450, Changzhou Hongyide Plastic Machinery Manufacturing Co., Ltd., Changzhou, China) and injected into a standard tensile part according to GB/T 1447-2005 [18], with a thickness of 2 mm, a width of 10 mm, and an effective length of 50 mm, in which the injection temperature of the injection molding machine was 280 °C, the injection pressure was 120 MPa, and the total injection time was 1 s.

### 2.3. Crystallinity 

Thermogravimetric analysis (TGA) and differential scanning calorimetry (DSC) tests were carried out on composites with different fiber contents using a simultaneous thermal analyzer (STA449F3, NETZSCH Instruments, Selb, Germany). For the TGA test, the samples were heated up to the test condition of 800 °C at the rate of 20 °C/min under the environment of nitrogen ventilation; a preheating cycle was required to the samples prior to the DSC test. Before the DSC test, a preheating cycle is required for the sample, i.e., the sample is heated from 50 °C to 300 °C at a rate of 20 °C /min under nitrogen, held for 5 min, and then lowered to 50 °C and held for 5 min in order to eliminate the thermal history of the sample. After the pretreatment, another thermal cycle of 50 °C–300 °C–50 °C was performed, during which the temperature was ramped up and down at a rate of 20 °C/min, and the experimental data were recorded.

### 2.4. Rheological Properties 

A capillary rheometer (RH2000, Malvern Panaco, Shanghai, China) was used for the rheological performance test, with a 1 mm diameter of the orifice mold, an L/D ratio of 16, five temperature points of 240 °C, 250 °C, 260 °C, 270 °C, and 280 °C, and a range of shear rates of 20–1000 s^−1^. In order to show the flow behavior and rheological properties of the melt at each shear rate under the description of the shear thinning behavior, the constitutive equations were fitted with the Bird–Carreau model. In order to describe the shear thinning behavior, the flow behavior, and the rheological properties of the melt at each shear rate, the present constitutive equations were fitted with the Bird–Carreau model.

### 2.5. Tensile Strength

Tensile strength testing was conducted in accordance with the GB/T 1040.2-2022 [19] standard. Tensile tests were carried out at room temperature using an electronic universal materials testing machine (ITW Group Instron, Shanghai, China). The testing parameters included a crosshead speed of 10 mm/min. 

### 2.6. SEM Observation 

After the specimens were sprayed gold, a field emission scanning electron microscope (Gemini 300, Carl Zeiss AG, Baden-Württemberg, Germany) was used for scanning analysis to observe the fiber orientation, distribution, and fracture behavior at the fracture.

**Figure 1:**
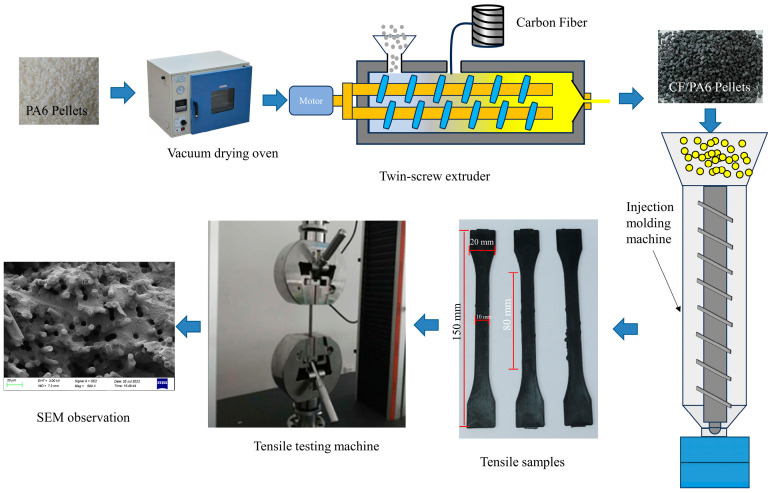
Experimental flow chart.

## 3. Results and Discussion

### 3.1. Crystallinity

**Figure 2:**
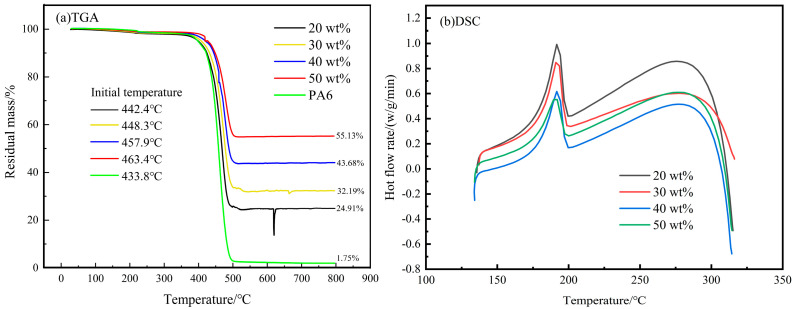
Simultaneous thermal analysis: (**a**) TGA and (**b**) DSC.

Figure 2 illustrates the results of thermogravimetric analysis (TGA) and differential scanning calorimetry (DSC) tests for CF/PA6 composites with varying carbon fiber contents.

From Figure 2a, the entire decomposition process of PA6 is concentrated in the 400 °C to 500 °C temperature range. However, depending on the carbon fiber content, the initial temperature of PA6 volatilization varies. For instance, at a carbon fiber content of 20 wt.%, the initial temperature is 442.4 °C, while at 50 wt.%, it increases by 21 °C. This suggests that a higher carbon fiber content enhances the thermal stability of the composites [13]. Figure 2b shows experimental data from the DSC curve during the cooling process from 300 °C to 135 °C. The upward curve indicates exothermic processes; that is, the curve undergoes an exothermic phase, then heat absorption leading to the melting peak, followed by another exothermic phase leading to the crystallization peak, and finally an absorptive process. Specific peak temperatures and melting enthalpies are detailed in Table 1. The table reveals that while the melting peak temperatures and crystallization peak temperatures are relatively similar across different samples, the difference between the temperatures, known as the crystallization window, decreases with an increase in carbon fiber content. Expanding the crystallization window not only allows for increased interlayer chains diffusion time but also aids in relieving residual stresses trapped within parts during injection molding, thereby enhancing the material’s mechanical properties. Conversely, a narrower crystallization window suggests that a higher carbon fiber content reduces the time available for interlayer chain diffusion, albeit with a relatively minor impact.

We utilize the melting enthalpy values from the table to calculate the crystallinity using the following formula provided below.

(1) Xc=ΔHmΔHf1−wf×100%

In the formula, $X_{c}$ is the crystallinity, $\Delta H_{m}$ is the melting enthalpy, ${\Delta H}_{f}$ is the reference enthalpy of fusion for 100% crystalline PA6 (taken as 188 J/g), and $w_{f}$ denotes the mass fraction of carbon fiber [20].

**Figure 3:**
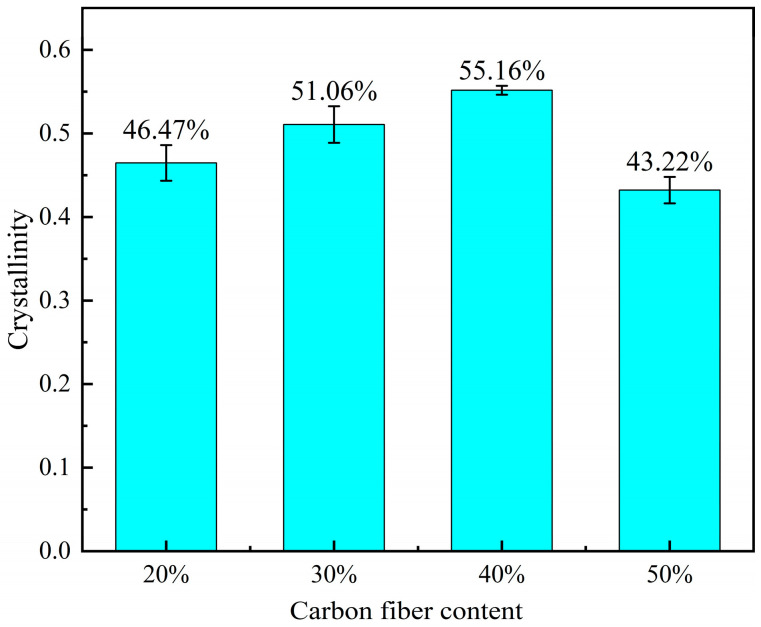
Crystallinity.

The computed results are depicted in Figure 3; the figure shows that the crystallinity increases and then decreases with the increase in carbon fiber content, peaking at 40 wt.%, where it reaches 55.16%. At 50 wt.%, crystallinity decreases, indicating that an excessively high carbon fiber content significantly hinders molecular chain diffusion, thereby impacting crystallization.

### 3.2. Steady-State Rheological 

**Figure 4:**
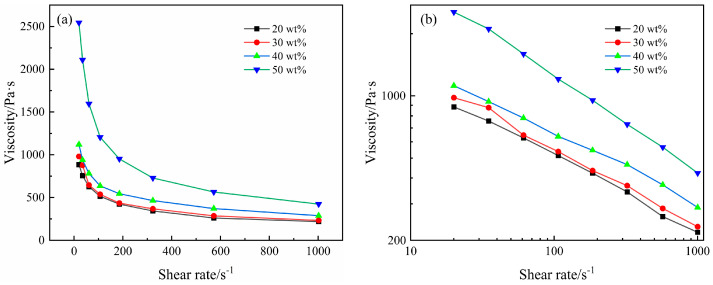
(**a**) Relationship between shear rate and viscosity. (**b**) The result of logarithmic operations on coordinates.

Behavior depicts the relationship between viscosity and shear rate for composites with varying carbon fiber content, tested at a temperature of 240 °C. Figure 4b is derived by applying logarithmic operations on the coordinates of Figure 4a. From the figure, it is evident that CF/PA6 composites behave as pseudoplastic fluids, where viscosity decreases with increasing shear rate. Furthermore, as can be seen from Figure 4b, the overall viscosity increases with a higher carbon fiber content [16]. At a carbon fiber content of 50 wt.%, the viscosity of the composites at 20 s^−1^ reaches 2544 Pa⋅s. This is three times higher than the viscosity of the composites containing 20 wt.% carbon fiber, measured under equivalent conditions. This contrasts with the trend observed in short-cut carbon fiber-reinforced polyamide 66 composites (SCF/PA66), where the apparent viscosity changes with carbon fiber content, initially decreasing and then increasing. This difference is primarily attributed to the lower thermal stability of PA66 at high temperatures. The self-lubricating nature of short-cut fibers and the orientation effect of fibers along the melt flow direction at certain shear rates significantly influence SCF/PA66, leading to a reduction in viscosity. Conversely, PA6, with its superior thermal stability and extrusion viscosity compared to PA66, presents a challenge for carbon fiber orientation. The predominant effects of carbon fiber’s high hardness and mutual accumulation impede the movement of molecular chains, consequently leading to an increase in viscosity.

**Figure 5:**
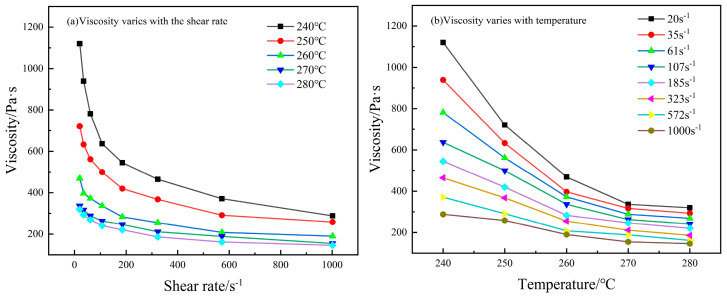
The viscosity of composites with a 40 wt.% carbon fiber content varies with temperature and shear rate.

Figure 5 illustrates the relationship between viscosity, temperature, and shear rate for composites with a 40 wt.% carbon fiber content. The figure reveals that the decrease in viscosity is a result of the combined effects of temperature and shear rate, demonstrating a trend akin to the viscosity curves of SCF/PA66. Near the melting point, the reduction in viscosity is largely due to the cut-off action between molecular chains. As the temperature elevates, the sensitivity of viscosity to shear rate diminishes. Similarly, at shear rates below 100 s^−1^, the decrease in viscosity is primarily ascribed to the rise in temperature intensifying the irregular movement of molecules, thereby enhancing intermolecular distance and flow capability. With the escalation in shear rate, the sensitivity of viscosity to temperature decreases. Furthermore, the impact of temperature and shear rate on viscosity has its limitations. As can be seen from the figure, significant viscosity changes with temperature mainly occur between 240 °C and 260 °C, while changes with shear rate mainly occur within the range of 0 to 400 s^−1^. Beyond these ranges, viscosity shows minimal variation.

To accurately determine the relationship between viscosity and shear rate, the Bird–Carreau model was used, along with the power law model, to fit the equations to the corresponding parameters at 240 °C [21].

Bird–Carreau model:

(2) η=η∞+η0−η∞(1+λ2γ2)n−12

Power law model:

(3) $$\eta =K\lambda^{n-1}$$

In the formula, $\eta_{0}$: zero-shear viscosity of the material, measured in Pa⋅s; $\eta_{\infty }$: viscosity of the material as the shear rate approaches infinity, measured in Pa⋅s; λ: relaxation time, controlling the shear rate of a fluid transitioning from a Newtonian stable plateau to power law behavior, measured in s; *n*: power law index (non-Newtonian coefficient), which governs the steepness of the power law curve in the high shear rate region; γ: shear rate, measured in s^−1^; K: consistency coefficient, measured in $\mathrm{Pa}\cdot s^{n}$.

**Figure 6:**
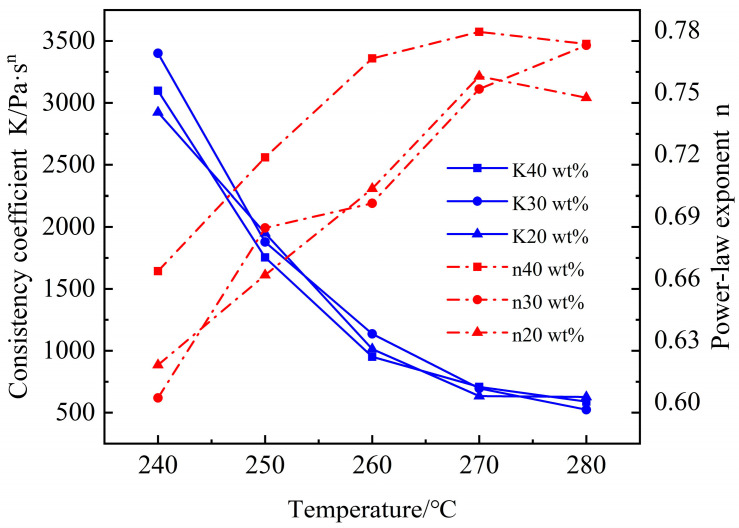
Effect of temperature on rheological parameters.

Table 2 and Figure 6 display the fitted results, where the power law index (*n*) symbolizes the sensitivity of viscosity to shear rate. A smaller *n* suggests a steeper power law curve, implying that viscosity is more sensitive to changes in shear rate. *K* is the consistency coefficient, which expresses the ratio of the settling velocity of solid particles at rest in water to the diameter of the particles and the viscosity of the melt. It primarily changes with temperature and material properties, directly reflecting the flow characteristics of the composites. A lower *K* value indicates a better flowability.

Table 2 illustrates the effect of carbon fiber content on rheological parameters at different temperatures. It is noteworthy that when the carbon fiber content is 40 wt.%, *n* reaches its peak value, whereas *K* diminishes by 300 $\mathrm{Pa}\cdot s^{n}$ relative to the 30 wt.% content. This indicates that, in comparison to 30 wt.% content, 40 wt.% content is more favorable for melt flow and displays a reduced dependency on shear. Consequently, it can be inferred that the distribution of CF in the composites with a 40 wt.% carbon fiber content is more uniform and the length is more concentrated and homogeneous. For composites incorporating 50 wt.% carbon fiber content, the corresponding *n* value is at its minimum, while *K* is at its maximum [22]. This implies that an excessive increase in carbon fiber content can decrease the flowability of the melt within the composites. At the same time, the number of molecular chains formed by microscopic entanglement of carbon fibers and PA6 is also greatly increased, resulting in an increased sensitivity to shear rate variations. As the shear rate increases from 20 s^−1^ to 200 s^−1^, a multitude of molecular chains undergo unraveling and breakage, causing the viscosity to decrease rapidly in response.

Figure 6 illustrates the effect of temperature variation on *n* and *K*. From the figure, it can be seen that the trend of *K* is similar to viscosity, decreasing with increasing temperature. This is primarily because higher temperatures intensify molecular motion, thereby improving the melt’s flowability. Conversely, *n* increases with temperature. This behavior is consistent with the rheological behavior of CF/PA6 composites, where at lower temperatures, the material is predominantly influenced by shear, resulting in a lower *n*. However, as the temperature rises, the influence of temperature becomes increasingly significant compared to shear, gradually reducing the impact of shear and thus increasing *n*.

### 3.3. Tensile Strength

**Figure 7:**
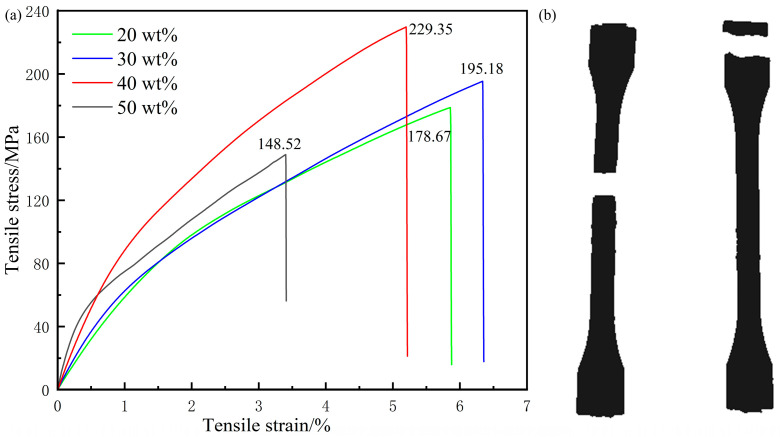
(**a**) Stress–strain curves of composites with different carbon fiber contents at room temperature and (**b**) fracture of samples with a 50 wt.% content.

Figure 7 depicts the stress–strain curves of composites with different carbon fiber contents at room temperature. The trend depicted in Figure 7a, characterized by an initial increase followed by a decrease, matches the variation in tensile strength observed in SCF/PA66 composites [23]. The difference is that for the PA66 matrix, the maximum tensile strength is achieved at a carbon fiber content of 30 wt.%. In contrast, for the PA6 matrix, the highest tensile strength of CF/PA6 composites is achieved at a carbon fiber content of 40 wt.%, reaching 229.35 MPa, which is 50.68 MPa higher than that of the 20 wt.% composites and 80.82 MPa higher than that of the 50 wt.% composites.

The tensile behavior of the composites with a 50 wt.% carbon fiber content differs from the previous three types of samples, as indicated in Figure 7b. The left fracture shows a central fracture of the tensile sample, which is a normal fracture form with a tensile strength of 148.52 MPa. On the right side, a clamping end fracture is presented with a tensile strength of only 117.28 MPa. This occurrence is attributed to two factors: first, the excessively high carbon fiber content increases brittleness, causing the clamping end to fracture under pressure during tension; second, increased carbon fiber content will cause the melt to condense faster, the first melt to be extruded will solidify quickly during injection molding, and due to the high carbon fiber content, the carbon fibers in the melt will not be sufficiently dispersed and oriented, resulting in defects in the sample part. Therefore, compared to other samples, the carbon fiber content of 40 wt.% is the most advantageous for enhancing the performance and strength of the composites.

### 3.4. SEM Topography

The fracture of CF/PA6 as a whole is similar to that of CF/PP. Due to the presence of carbon fibers, which enable the expansion of mutation cracks from the tips of the fibers, the composites basically fail by brittle fracture after the addition of carbon fibers, but there are also some cases of plastic deformation of the PA6 matrix. As the carbon fiber content increases, the mobility and migration of polymer chains are hindered, resulting in reduced plasticity of the composites and a decrease in strain at fracture, accompanied by an increase in Young’s modulus [24].

**Figure 8:**
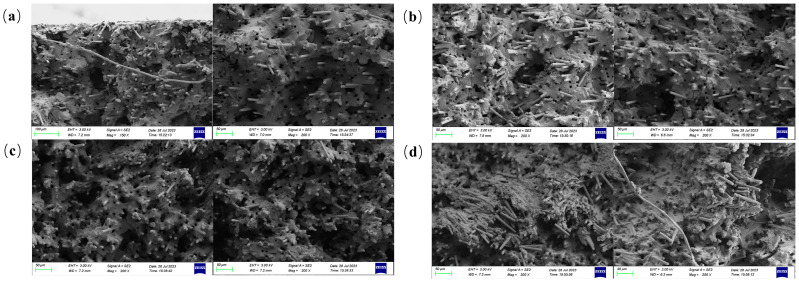
SEM photos of tensile specimen of composites with different carbon fiber content. (**a**) 20 wt.% (**b**) 30 wt.% (**c**) 40 wt.% (**d**) 50 wt.%.

Figure 8 shows SEM images of the tensile cross-section of samples with different carbon fiber contents. The fracture surface of the 20 wt.% sample, due to the low carbon fiber content, shows a good flowability; each carbon fiber is wrapped by a large amount of matrix material and the carbon fiber orientation is primarily along the extrusion direction and is more uniformly dispersed. However, due to the small shear strength, there are unsheared carbon fibers as well as some longer carbon fibers that are perpendicular to the extrusion direction. Compared to the fracture surface with a 20 wt.% carbon fiber content, the fracture surface with a 30 wt.% exhibits a relatively disordered distribution. Most carbon fibers are oriented at certain angles to the extrusion direction, and fiber stacking becomes evident as the carbon fiber content increases. Upon further increasing the carbon fiber content to 40 wt.%, the fibers in the fracture surface align significantly along the extrusion direction, with some stacking observed, yet the majority of fibers are relatively evenly distributed. This is because the rotational movement of the screw during processing facilitates the orientation and dispersion of the carbon fibers in composites, particularly those with a carbon fiber content of 40 wt.%. Consequently, compared to other specimens, the fibers in the 40 wt.% sample exhibit a tighter integration with the matrix, resulting in stronger interfacial forces. At a 50 wt.% carbon fiber content, excessive fiber accumulation perpendicular to the extrusion direction occurs on the cross-section. This prevents adequate matrix infiltration of the fibers, which limits the mechanical performance potential of the carbon fibers and significantly reduces the overall interfacial strength.

**Figure 9:**
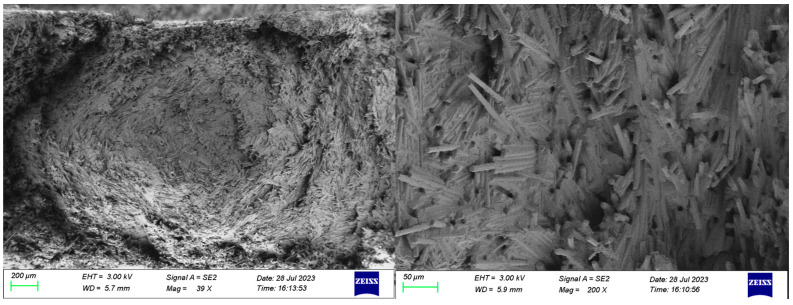
Fracture topography of a sample with a 50 wt.% carbon fiber content.

Figure 9 illustrates the fracture surface of the tensile specimen with a 50 wt.% carbon fiber content [25]. It is evident that the carbon fibers are predominantly aligned in a helical pattern perpendicular to the extrusion direction. Due to extensive fiber accumulation, the impregnation rate is low, which results in insufficient bonding with the matrix and a lack of interfacial strength. Consequently, under tensile stress, a brittle fracture occurs where the matrix fails cohesively, which leads to the observed cohesive failure of the matrix.

Variations in fiber length and distribution due to different carbon fiber contents lead to corresponding changes in the fracture modes of the injection-molded parts. Upon examining the cross-section in Figure 8, it is observed that the fracture modes for samples with 20, 30, and 40 wt.% carbon fiber contents are predominantly characterized by fiber pull-out and fiber fracture. However, fiber pull-out predominates overall. As the carbon fiber content increases, more fibers bond with the resin, which leads to instances where the resin matrix is pulled out along with the fibers. In contrast to samples with other contents, the sample with a 40 wt.% carbon fiber content exhibits a different pattern of fiber pull-out.

**Figure 10:**
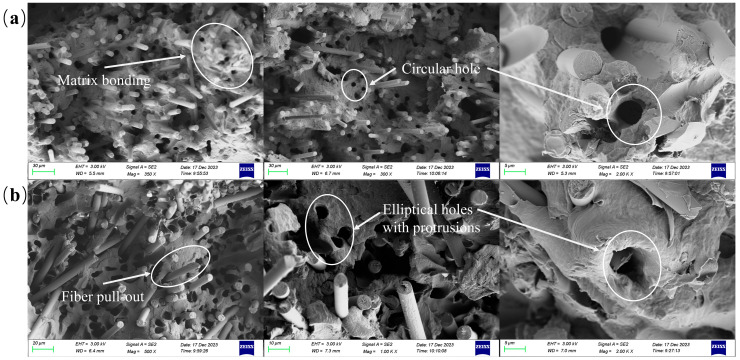
Fracture of tensile specimen of composites with a 40 wt.% carbon fiber content. (**a**) Core fiber breakage; (**b**) Edge fiber breakage.

**Figure 11:**
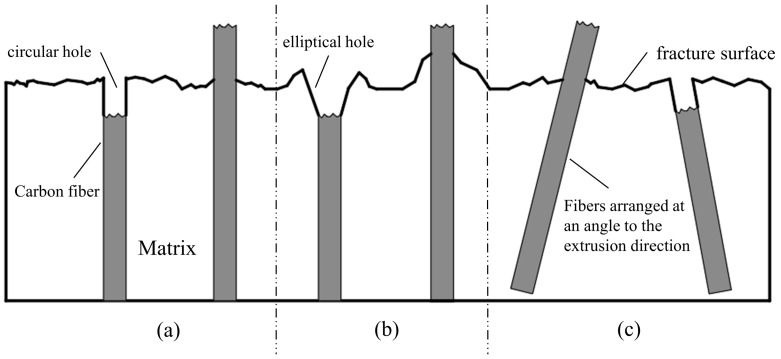
Fiber fracture model diagram: (**a**) Core fiber breakage; (**b**) Edge fiber breakage; (**c**) Fracture of fibers arranged at an angle along the extrusion direction.

Figure 10 depicts the fracture behavior of the composite specimen with a 40 wt.% carbon fiber content. Figure 11 shows the model of the three fiber pull-out fracture cases. Figure 10a shows the form of carbon fibers being pulled out from the core during the fracture of the tensile sample. It can be seen from this figure that the matrix holes are very regular circles, and there is no resin adhesion on the surface of the protruding carbon fibers, which is consistent with Figure 11a. This indicates a weak interaction between the resin in the core and the carbon fibers, characterized by a low interfacial strength, which easily breaks under tensile stress. This pattern is consistent with the fiber pull-out observed in specimens with other carbon fiber contents.

Figure 10b shows the pull-out of carbon fibers at the edge during fracture, where the holes are depicted as irregular ellipses with raised shapes, as illustrated in Figure 11b. This suggests that during fiber pull-out, adhesive bonding occurs between the fibers and the resin, indicating a significantly stronger interfacial strength at the periphery compared to the core. This interaction causes deformation in the matrix upon fiber pull-out. Therefore, due to these interfacial characteristics, the tensile strength of the sample with a 40 wt.% carbon fiber content is the highest among specimens with different contents. 

## 4. Conclusions

In summary, four pellets with different carbon fiber content were prepared using a twin-screw extruder. The crystallization and rheological properties of the materials were then investigated using a simultaneous thermal analyzer and a capillary rheometer. The results show that increasing the carbon fiber content enhances the thermal stability and narrows the crystallization window of CF/PA6. The viscosity increases with higher carbon fiber content and decreases with elevated temperature and shear rate. Subsequently, the strength of the materials was tested by injection molding the particles into tensile samples, and the fracture morphology was observed using SEM. The results show that the samples with a 40 wt.% carbon fiber content have a good fiber dispersion and orientation and a high interfacial bond strength, resulting in the best tensile strength of the samples. When the content is 50 wt.%, large injection defects occur at the clamping end due to high content, high brittleness, and large fiber stacking. The arrangement direction of the fibers is perpendicular to the extrusion direction, which leads to large tearing of the substrate during stretching. Therefore, CF/PA6 with a 40 wt.% carbon fiber content is the optimal combination. Overall, these studies are expected to contribute to the development and modification of CFRP materials, thereby increasing their prospects for industrial applications.

## Figures and Tables

**Table 1:** Differential scanning calorimetry data of CF/PA6 composites with different mass fractions of carbon fiber (CF).

Mass Fraction of CF/wt.%	T_1_/°C	T_2_/°C	T_3_/°C	$\Delta H_{m}$
20	200.91	191.59	9.32	69.89
30	200.10	190.95	9.15	67.19
40	199.62	191.63	7.99	62.22
50	198.61	190.65	7.96	40.63

Notes: T_1_ is the melting peak temperature; T_2_ is the crystallization peak temperature; T_3_ is the crystallization window; $\Delta H_{m}$ is the melting enthalpy.

**Table 2:** Rheological parameters for different CF contents.

	20 wt.%	30 wt.%	40 wt.%	50 wt.%
$\eta_{0}$	1048.33	1241.84	1655.35	3333.05
λ	0.06	0.07	0.15	0.07
n	0.62	0.60	0.66	0.51
K	2923.58	3401.05	3098.07	11,600.35

## Data Availability

The testing and analysis data used to support the findings of this study are included within the article.
